# You can't start a fire without a spark: Extracellular ATP triggers systemic ROS wave after local leaf wounding

**DOI:** 10.1093/plphys/kiac191

**Published:** 2022-04-27

**Authors:** José Manuel Ugalde

**Affiliations:** INRES—Chemical Signalling, University of Bonn, Bonn 53113, Germany

Plants are constantly challenged by wind, freezing temperatures, heat, drought, herbivores, and pathogens. All these stresses produce mechanical wounding in locally affected tissues, which causes the collapse of different subcellular structures and disruption of the plasma membrane. When the plasma membrane loses its integrity, a cocktail of damage-associated molecular patterns is released from the cytosol, transducing an “alert signal” to the neighboring cells (Vega-Muñoz et al., 2020). Among the released molecules are glutamate (Glu) and the nucleotide adenosine triphosphate (ATP, or external adenosine triphosphate [eATP] when it is extracellular). eATP and Glu released from the damaged cells are sensed by adjacent cells via membrane-bound PURINORECEPTOR 2 KINASEs (P2Ks, also known as DOESN’T RESPOND TO NUCLEOTIDES1 (DORN1) and GLUTAMATE-LIKE RECEPTORS (GLRs), respectively (Figure 1A). Upon ligand recognition, these receptors trigger wound-induced signal transduction pathways, such as an increase in reactive oxygen species (ROS) production and upregulation of stress-related genes (Chen et al., 2017; Toyota et al., 2018; Vega-Muñoz et al., 2020).

**Figure 1:**
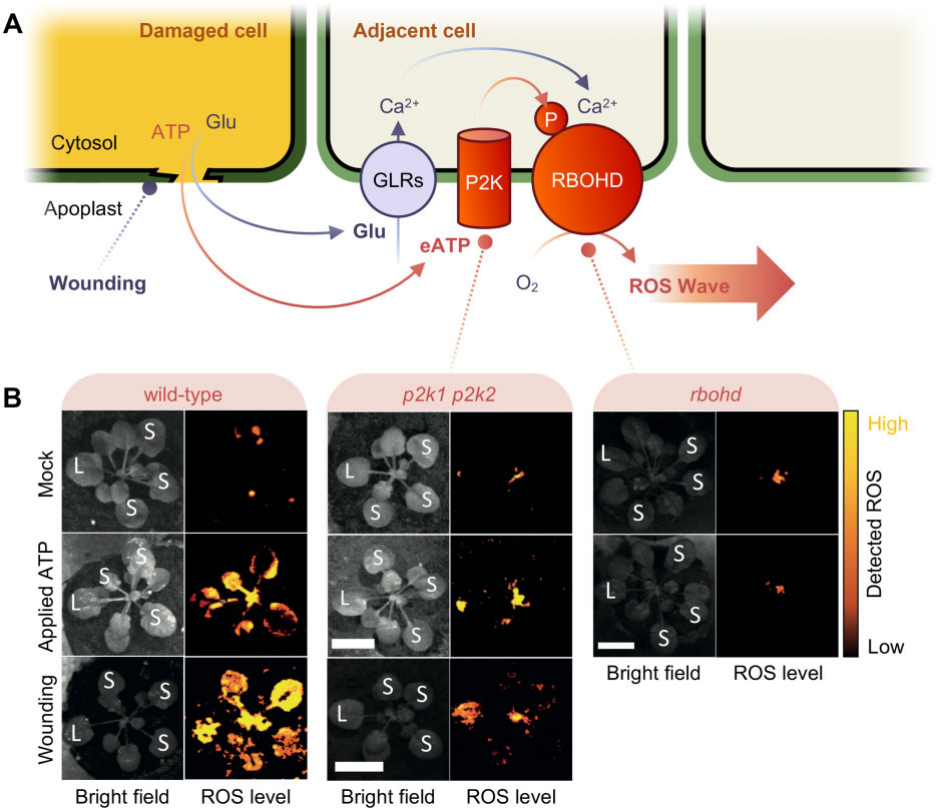
eATP is sufficient to trigger a P2K- and RBOHD-dependent systemic ROS wave. A, Upon mechanical wounding, disruption of the plasma membrane enables the release of Glu and eATP, among others, to the apoplast. Glu and eATP are sensed by GLRs and by P2K, respectively. Both GLRs and P2K contribute to the activation of the respiratory burst oxidase homolog D, RBOHD, which enhances apoplastic ROS production and enables the ROS wave. B, ROS production in Arabidopsis rosettes from wild-type, *p2k1 p2k2* double mutant, or *rbohd*. ROS levels are followed in real time as the oxidation of dichlorofluorescein after ATP treatments or wounding on a local leaf (L). The intensity of detected ROS is represented as a false coloring scale from black to yellow throughout the nontreated systemic tissue (S). Unlike wild-type plants, the ATP-induced ROS wave is almost completely abolished in the double mutant *p2k1 p2k2* and in the *rboh* mutant. Images from (B) extracted from Myers et al. (2022).

Depending on how severe the damage is, signals can be propagated from the wound site to the rest of the plant, firing response mechanisms in tissues that were not subjected directly to wounding, in a process called systemic wound response (SWR; Walker-Simmons et al., 1984; Vega-Muñoz et al., 2020). Even after nearly 40 years, identifying the signals linked to SWR is still a work in progress. Some of the signals that have been implicated in SWR are rapid changes in membrane potential, Glu, phytohormones, and ROS and calcium (Ca^2+^) waves. Released Glu from wounded cells triggers systemic Ca^2+^ and ROS waves on unwounded tissues through the action of GLRs and the RESPIRATORY BURST OXIDASE HOMOLOG D (RBOHD; Toyota et al., 2018). ATP has also been shown to trigger local production of ROS during wounding via the purinoreceptor 2 kinase (P2K) that senses eATP and phosphorylates RBOHD, promoting ROS production (Chen et al., 2017). However, the role of eATP in the SWR is not yet established.

In this issue of *Plant Physiology*, Myers et al. (2022) report how eATP can be locally detected upon wounding and how eATP is sufficient to trigger a systemic ROS wave in unwounded leaves. Furthermore, the authors demonstrate that, as previously shown for the local response, the eATP-induced SWR depends on the membrane-bound eATP-receptor P2K and RBOHD. To detect eATP and ROS, the authors used the IVIS Lumina S5 platform, which allowed them to measure ROS dynamics in real time in whole Arabidopsis (*Arabidopsis thaliana*) plants exposed to different stress conditions (Fichman et al., 2019).

Since eATP triggers local ROS production via P2K-dependent phosphorylation of RBOHD (Tanaka et al., 2010; Chen et al., 2017), the authors tested ROS production after treatments with a stable ATP form, βγmeATP. Arabidopsis plants were sprayed with 2,7-dihydrodichlorofluorescein diacetate (H_2_DCF-DA), and ROS levels were measured continuously in the whole rosette as dichlorofluorescein oxidation over time. Their results show that treatments as low as 100-nM ATP on a leaf can trigger the systemic ROS wave 10–30 min later. Furthermore, they demonstrate that following ATP application, the ROS wave in nontreated systemic leaves was suppressed in lines deficient in P2K, such as the double mutant *p2k1 p2k2* and the *rbohd* mutant (Figure 1B). These results indicate that ATP application is sufficient to initiate ROS waves systemically.

Finally, the authors established the importance of the eATP receptor, P2K, in the SWR. Using the same setup, they showed that after local wounding, both the ROS wave and the accompanying induction of defense genes in nonwounded systemic leaves were inhibited in the single and double *p2k* mutants. Since ROS suppression was not complete, the authors explored if the ROS wave depended on additional wound-related signals, such as Glu. Interestingly, when ATP and Glu were simultaneously applied, the ROS signal was not additive, suggesting that ATP and Glu operate in the same pathway.

In summary, Myers et al. (2022) showed that eATP acts as a spark that triggers the systemic ROS wave and that this response depends on the eATP receptor P2K and RBOHD. It would be interesting to evaluate further if eATP and Glu share the same signaling pathway. As mentioned by the authors, this idea could be tested by assessing the eATP-induced systemic ROS wave in the double mutant of the glutamate-like receptor *grl3.3 grl3.6*, which also is defective in triggering systemic signaling upon wounding (Fichman and Mittler, 2021). Furthermore, the imaging setup used by the authors represents an exciting opportunity for the use of genetically encoded biosensors by increasing resolution in the dynamic changes of different cellular components and allowing us to explore differences in real time with a subcellular resolution.

*Conflict of interest statement*. None declared.

## References

[kiac191-B1] Chen D , Cao Y, Li H, Kim D, Ahsan N, Thelen J, Stacey G (2017) Extracellular ATP elicits DORN1-mediated RBOHD phosphorylation to regulate stomatal aperture. Nat Commun 8: 2265 29273780 10.1038/s41467-017-02340-3 PMC5741621

[kiac191-B2] Fichman Y , Miller G, Mittler R (2019) Whole-plant live imaging of reactive oxygen species. Mol Plant 12: 1203–1210 31220601 10.1016/j.molp.2019.06.003

[kiac191-B3] Fichman Y , Mittler R (2021) Integration of electric, calcium, reactive oxygen species and hydraulic signals during rapid systemic signaling in plants. Plant J Cell Mol Biol 107: 7–20 10.1111/tpj.15360 34058040

[kiac191-B4] Myers RJ , Fichman Y, Stacey G, Mittler R (2022) Extracellular ATP plays an important role in systemic wound response activation. Plant Physiol **189**: 1314–1325 10.1093/plphys/kiac148 PMC9237675 35348752

[kiac191-B5] Tanaka K , Gilroy S, Jones AM, Stacey G (2010) Extracellular ATP signaling in plants. Trends Cell Biol 20: 601–608 20817461 10.1016/j.tcb.2010.07.005 PMC4864069

[kiac191-B6] Toyota M , Spencer D, Sawai-Toyota S, Jiaqi W, Zhang T, Koo AJ, Howe GA, Gilroy S (2018) Glutamate triggers long-distance, calcium-based plant defense signaling. Science 361: 1112–1115 30213912 10.1126/science.aat7744

[kiac191-B7] Vega-Muñoz I , Duran-Flores D, Fernández-Fernández ÁD, Heyman J, Ritter A, Stael S (2020) Breaking bad news: dynamic molecular mechanisms of wound response in plants. Front Plant Sci 11: 610445 33363562 10.3389/fpls.2020.610445 PMC7752953

[kiac191-B8] Walker-Simmons M , Holländer-Czytko H, Andersen JK, Ryan CA (1984) Wound signals in plants: a systemic plant wound signal alters plasma membrane integrity. Proc Natl Acad Sci USA 81: 3737–3741 16593475 10.1073/pnas.81.12.3737 PMC345294

